# Structure, Process, and Outcomes of Liberian National Nursing and Midwifery Curricular Revisions

**DOI:** 10.5334/aogh.3248

**Published:** 2021-10-08

**Authors:** Cecelia C. Kpangaala-Flomo, Mary W. Tiah, G. Clinton Zeantoe, Humphrey Gibbs Loweal, Rita Florence Matte, Sodey C. Lake, Susan D. Altman, Maria Mendoza, Tanya Tringali, Kerry Stalonas, Lloyd Goldsamt, Renata Kurz, Lily Zogbaum, Robin Toft Klar

**Affiliations:** 1 Liberian Board for Nursing and Midwifery, LR; 2 United Methodist University, LR; 3 Phebe Hospital and School of Nursing, LR; 4 New York University Rory Meyers College of Nursing, US

## Abstract

**Background:**

The Republic of Liberia has had major disruptions to the education of its health care cadres. Post Ebola, the Resilient and Responsive Health Systems (RRHS) initiative began a new era of capacity building with the support of PEPFAR and HRSA. Nursing and Midwifery serve as the largest healthcare cadres in Liberia. The national nursing and midwifery curricula were overdue for the regulated review and revisions.

**Methods:**

The Science of Improvement was used as the framework to accomplish this multilateral activity. The Institute for Healthcare Improvement’s (IHI) stages of improvement included: 1) Forming the team, 2) Setting the aims, 3) Establishing measures, 4) Selecting measures, 5) Testing changes, 6) Implementing changes, and 7) Spreading changes. These stages served as the blueprint for the structures and processes put into place to accomplish this national activity.

**Findings:**

The RN, Bridging, and BScM curricula all had redundant content that did not reflect teaching pedagogy and health priorities in Liberia. Courses were eliminated or reconfigured and new courses were created. Development of Nursing and Midwifery Curricular Taskforces were not as successful as was hoped. Two large stakeholder meetings ensured that this was the curricula of the Liberian faculty, deans and directors, and clinical partners. Monitoring and evaluation tools have been adopted by the Liberian Board for Nursing and Midwifery to serve as another improvement to check that the new curricula are being implemented and to identify gaps that may require future cycles of change for continued quality and improvement.

**Conclusions:**

Developing trust among the multilateral partners was critical to the success of this activity. Networks have been expanded, and a proposed pilot with the Ghana Board of Nursing and Midwifery and the US academic partner will examine the feasibility of implementing electronic licensing examinations for nurses and midwives.

## Background

The health of a county greatly depends on the competency and capacity of its healthcare workers. The World Health Organization (WHO) provides strategies to support the advancement of human resources for health through 2030 [1]. Transforming the competencies of future cadres of nurses and midwives aims to improve the quality of care provided to future patient populations. Additionally, these transformations should target the increase of resilience and responsiveness of health care workers to adapt to constantly changing environments; civil disputes, novel diseases, disasters, demographics, and dissemination of evidence. This paper shares the context of nursing and midwifery education in Liberia and the structures and processes that supported the outcome of three revised curricula.

Formal nursing and midwifery education began in Liberia around 1922. Nursing’s minimal education results in earning a diploma. There are currently articulation programs for registered nurses with a diploma to earn a Bachelor’s of Science in Nursing (BScN) degree and there are several accredited direct entry BScN programs. Midwifery education has evolved from a certificate program to become a certified midwife (CM) to a registered midwife (RM) earning a diploma or an associate’s degree. Certificate programs were dissolved, with the last cohort taking their licensing exam in 2012. There are three curricula currently in use that were the focus of this project: 1). Pre-licensure registered nurse (RN), 2). CM to RM bridging (Bridging), and 3). Bachelor of science in midwifery (BScM).

This national nursing and midwifery curricular revision initiative had its beginnings with the development of the Liberia Health Workforce Program FY 2015–2021 [2] prompted by the Ebola Virus epidemic in 2014. This epidemic, more than the decimation of a large percentage of its population due to two civil wars, highlighted the lack of a competently skilled workforce. The current SARS CoV 2 pandemic has solidified the importance of a competent health workforce that supports the expansion of a resilient and responsive health system.

## Methods

This initiative was informed by the Science of Improvement, which brought together academicians and clinicians to examine the current nursing and midwifery curricula and revise them, to close the gap between academia and clinical care and to educate nurses and midwives to be fit for practice [3]. We actualized the science of improvement using the Institute for Healthcare Improvement’s (IHI) Model for Improvement [4], which includes the following stages: 1). Forming the team, 2). Setting the aims, 3). Establishing measures, 4). Selecting measures, 5). Testing changes, 6). Implementing changes, and 7). Spreading changes.

## Forming the team

Two large cohorts made up this multilateral team. The Liberia cohort consisted of all members of the Liberian Board for Nursing and Midwifery (LBNM). The LBNM is made up of deans and directors of all accredited nursing and midwifery schools, along with clinical and community partners. The LBNM Secretariat played a direct role in ensuring all standard operating procedures were implemented for this national curricular review and revision. The United States (US) academic institutional cohort was made up of nursing and midwifery faculty, a monitoring and evaluation specialist, and an operations specialist.

### Setting the aims

The goal of this initiative was to revise the national midwifery curricula and nursing curriculum. The aims were broken down to mirror the steps in the LBNM process for curricular revisions.

Aim One was to share the current curricula with the US team. Additionally, other documents were shared to provide data on identified gaps in knowledge, both at the licensing level—with recent aggregate test scores of the schools of nursing and midwifery—and a task analysis [5] that identified current roles nurses and midwives serve and where they first learned about these roles. US nursing and midwifery faculty analyzed these data and established guiding principles for conducting a 360° review of each curriculum.

### Establishing Measures

Measures established for review and revisions of the nursing curriculum were guided by Billings and Halstead framework [6] which examines the interrelationships of curricular domains, which include: Mission/Vision/Values, Professional Values, Philosophy, Personal Values of Faculty, Conceptual Frameworks, End of Program Competencies, Curriculum Design, and Courses. To contextualize this curricular review and revisions, we added two subdomains to further define the Liberian nursing education environment. The first subdomain included: Who are our learners, Job descriptions, and Country Health Priorities, and fell under the domains of End of Program Competencies and Curriculum Design. The second subdomain of Resources and Facilities Needed added further context to Learning Environments, which falls under the domain of Courses.

The measures established for the Bridging curriculum and BScM curriculum originated from the International Confederation of Midwifery (ICM) global standards and core competencies [7] and the Liberian Task Analysis [5]. Measures included Humanistic and Scientific Principles, Promotion of Growth, Self-Sufficiency, Life-long and Problem-based Learning, as well as Critical Thinking and Group Interactions.

### Selecting Measures

In addition to measures identified under establishing measures for the review and revision of the three curricula, we also selected measures for the first LBNM stakeholder meeting. We developed an updated version of the Liberian Task Analysis to get a picture of the participants impressions of current task acquisition. We also developed a short survey to assess the stakeholder participants ideation on the development and ownership for Continuing Professional Development courses.

Standard LBNM processes for Curricular Revisions were also determined. The LBNM Secretariat set up a plan and informed the Board that the curriculum had reached a point for review and update. A workplan was developed and approved by the LBNM Registrar and Chairperson. Since funding was already secured as an approved activity for the RRHS Liberian Workforce grant, our US academic lead partners and LBNM Secretariat discussed the curricular revision landscape. The landscape included the amount the outside partner can contribute, sustainability of the full revision process, and level of commitment. If the total process could not be completed, then agreements were made that LBNM could finish up those areas not funded by the outside partner.

## Results

Results are aligned with the IHI Science of Improvement stages of Testing changes and Implementing changes. These changes involve both the actual review of the curricula with initial revisions by the US academic partner and the follow up changes that occurred in two LBNM stakeholder meetings.

### Testing Changes

The US academic institution’s faculty completed a preliminary review of the nursing curriculum at the beginning of the new 2017 (September) semester with a presentation to the Board Secretariat in December 2017. Additional review and recommendations for revised curricula were completed by the US academic institutional partner from January through June 2018, and the midwifery curricular review was initiated.

US academic nursing curriculum review began with a full curriculum mapping process. This was a challenging task since the original curriculum has many program outcomes. Nevertheless, we completed the task but did not find much practical value to it. There was a discussion whether we should consolidate the original program outcomes into more manageable numbers but abandoned the idea due to not having enough time to get feedback from the group.

The midwifery curricular review began by mapping both the Bridging and BScM curricula in their entirety to the ICM standards and the core competencies (General, Specific to Pre-pregnancy and Antenatal Care, Specific to Care during Labor and Birth, and Specific to Ongoing Care of Women and Newborns) and midwifery workforce tasks identified in the Task Analysis.

The testing changes preliminary recommendations were shared with the LBNM Secretariat in December 2017. Additional review and recommendations for revised curricula were completed from February through May 2018 in preparation for the first LBNM Curricular Review Stakeholder Workshop, held June 13–15, 2018, where the implementing changes cycles began.

The LBNM Secretariat and the US academic institution collaborated on the processes and objectives for the first Curricular Revisions LBNM Stakeholder meeting. This was a two-and-a-half-day meeting from June 13–15, 2018. Meeting objectives included: 1). Disseminate Nursing and Midwifery curricular review process and recommendations, 2). Identify topics for further in-depth review by stakeholders, 3). Initiate the adaptation of Nursing and Midwifery curricula in Liberia, and 4). Create two taskforces (nursing and midwifery) to finalize curricula.

Nursing curricular review prompted revisions to all syllabi that were determined to be relevant to nursing education. Several courses were eliminated due to redundancy and lack of relevance to the current Liberian academic context. Recommendations were developed and put forth to the full LBNM first Stakeholder meeting, held over three days in June 2018 included activities listed in ***Table 1***.

**Table 1:** Nursing Curricular Review’s Recommended Changes.


The post-Ebola curriculum: Need to identify competencies, i.e., knowledge, skills, and attituded (KSA) related to care of patients with Ebola

Improve alignment between course outcomes and course content

Level content to ensure building knowledge from simple to complex

Strengthen the psychosocial content of the curriculum

Revise non-clinical courses to build foundation for clinical courses

Identify skills competencies for nurse related Information Communication Technology

Use the nursing process as the framework in teaching diseases

Include a course in Nursing Care of the Elderly

Clarify clinical hours for clinical courses


Midwifery curricular revisions after review included the elimination of six courses in the Bridging Curriculum due to redundancy. Two courses were extensively redesigned, and three new clinical courses were created. The clinical courses allow for more hands-on practice. Clinical Affiliation I includes three weeks in the lab practicing physical exam skills, includes a skills check, and is followed by an experience in the Outpatient setting, with an emphasis on assessment, diagnosis and treatment of primary care and tropical and infectious diseases. Clinical Affiliation II includes six weeks of gynecology in both inpatient and outpatient settings including access to surgical cases, six weeks in both psych/mental health, inpatient and outpatient settings, and four weeks in an emergency room or other appropriate setting for Emergency and Disaster Preparedness. Also included are three objective structured clinical examinations (OSCEs) in the lab in each of the content areas. Clinical Affiliation III includes antepartum, intrapartum, postpartum, and newborn care. Three OSCEs in the lab should occur in content areas including labor and birth, postpartum, and newborn resuscitation.

Recommended changes to the BScM curriculum resulted from the same comprehensive review process as was conducted for the Bridging curriculum. Curricular changes included the elimination of 18 of 34 courses and reducing this program from six to four semesters. These courses were either deemed to be redundant or of limited relevance. A number of courses were considered essential in order to improve literacy, numeracy, critical thinking and professional development. A statistics course was added to the curriculum as it was deemed essential by all faculty and stakeholders. Some courses, including Research Methods, Embryology and Genetics, Pathophysiology of the Reproductive System, Teaching Methodology, and Senior Seminar/Practicum were extensively redesigned to assure adequate exposure to the necessary content.

The midwifery model of care was threaded throughout the curriculum in an effort to deemphasize the more medical model and nursing framework previously used. Concepts such as shared decision making, patient teaching and advocacy were highlighted in order to better align with the current ICM competencies. Credits were streamlined to be consistent with curricula in all three programs.

### Implementing Changes

The first LBNM Curricular Review Stakeholder Workshop included 45 members of the LBNM as well as representatives from UNICEF, UNFPA, ACCEL, JHPIEGO, HRSA, and the US nursing institution team.

The first implementing change cycle began with the presentation of all three curricula reviews and revisions. The two US academic institution nursing faculty presented the nursing curriculum revisions with rationales for each change. One US academic institution midwifery faculty presented both the Bridging and BScM curricula revisions with rationales for change. Following these presentations, attendees broke into small working groups to analyze and critique the recommendations. Objectives were carefully examined and where there was clear consensus among stakeholders in support of a particular change, that change was made. Where there were differing opinions among stakeholders, courses were reviewed to assure competencies were adequately covered. From here, a decision was made to cut or create a new course. Likewise, where a deficiency was noted, new content or a specific teaching/learning strategy was implemented. Pedagogical activities were integrated to support more complex critical thinking skills, such as those involved in developing a differential diagnosis and documentation skills such as SOAP notes.

At the end of this process two task forces were formed (Nursing and Midwifery), with volunteers assigning themselves to a respective task force. The purpose of the task forces was to support another cycle of change as the US institutional faculty brought back the information from the small working groups’ feedback.

In addition to presentation of curricula and feedback at this workshop, this first implementation change cycle included presentations from two clinical sites for a perspective on how they work with the schools as clinical sites, and to discuss fit for practice strengths and gaps. A follow-up Task Analysis was conducted by all LBNM members present to determine any shifts in tasks since the previous Task Analysis conducted in 2009–2010.

Time away from the classroom and clinical setting can create a burden, so collaborating partners used the time and resources that brought these stakeholders together to support additional LBNM activities. During this meeting we gathered information from the participants regarding how they saw the implementation of the newly adopted requirements by the LBNM that all nurses and midwives up for license renewal would need to demonstrate participation in continuing professional development (CPD) courses/activities. Questions asked were: 1) How do you determine the priority areas for CPD in your county or your facility? 2) Who is the best stakeholder or provider of CPDs? 3) What are some of the best methods of CPD administration? (i.e., Hands on, online, DVD, mobile phone, etc.) and 4) Do you have training courses now that you would like to get accredited for CPD credit?

The second test of Implementing Change was the work of the Nursing and Midwifery Curricular Task Forces. They were sent a list of all members of each task force. Each volunteer member (18 Nursing and 11 Midwifery) was assigned to a set of specific syllabi to add their feedback on the next round of revisions. This attempt at an additional test of implementing change was not well adopted. The US academic institutional partners came to understand that, once members of the LBNM disperse back to their respective institutions, it is difficult to reengage due to work and internet access burdens. The feedback we did receive was integrated into the next round of revisions.

The third cycle of Implementing Changes occurred with the second LBNM Curricular Revisions Stakeholder meeting. This meeting was conducted in Monrovia over three days from February 18–20, 2019, with the ultimate goal to review, validate, and adopt the three curricula for a five-year period. Specific objectives included: 1) Review the curricula revision work process conducted by the nursing and midwifery taskforces from June 2018 through February 2019; 2) Presentation of individual curriculum revisions by US academic institution faculty and taskforces; 3) Provide full LBNM feedback and review of curricular revisions through smaller working group meetings and report out to the full board; 4) Validate curricula; 5) Draft resolution for acceptance of the LBNM validation of all three curricula; and 6) Sign resolution for acceptance of the three curricula. All objectives were met during these three days.

Our fourth cycle of Implementing Changes involved making final edits to the curricula over the next six months. This was an important and tedious step in assuring the quality of the final electronic and hard copies of each curriculum. The country context was kept in mind during the editing of the existing syllabi as well as during the creation of the new ones.

Once the US academic institution faculty were done with the syllabi editing and creation, each course syllabus was sent to our Global Initiatives administrator for formatting and collating. During this phase of the process, which took several weeks to complete, special attention was given to proofreading and standard formatting practices to ensure writing and formatting consistency. The course credits were also reviewed to certify that they were in line with the course workload and with Liberia’s guidelines for course credit allocation. If the number of credits did not comply with these requirements, the credits were revised accordingly. The credit calculations were based on 14 instructional weeks and the following formulas were used: 1 didactic hour per week = 1 credit hour and 3 clinical (including lab) hours per week = 1 credit hour. Electronic versions were released to LBNM members in August 2019. The US academic institution partner also made provisions for hard copy distributions of the curricula to be made along with the curricula dissemination activities that were planned for December 2019.

The fifth cycle of Implementing Changes occurred with the Dissemination of Revised Curricula, which occurred in December 2019. A multilateral team of two midwifery—BNM Secretariat Midwifery Director and visiting midwifery faculty who has experience teaching in three schools in Liberia—and two nursing (Dean of one of Liberia ‘s universities and US academic institution principal investigator) members drove to identified schools of nursing and midwifery located in Lofa, Bong, Margibi, Nimba, and Montserrado counties. A formal presentation was given to faculty, students, and administrators at each school. All participants took advantage of the question-and-answer period. Students’ most common concern expressed was how these newly revised curricula would impact the progression of current students. Faculty members most common concern was whether they would be receiving faculty development to implement the curricula. An official handover of the new curriculum concluded each school dissemination event. The remaining schools of nursing and midwifery had their dissemination event performed by the LBNM Secretariat over the next several months.

### Spreading Changes

During the dissemination phase, one Dean shared that their school had already implemented the newly developed Nursing Care of the Aging Population course. Prior to the national dissemination of the curricula in December 2019, the Liberian Board for Nursing and Midwifery approved the implementation of the curricula by all training institutions in Liberia. Upon receipt of the information at the United Methodist University, the Dean of the School of Nursing and Midwifery immediately called a strategic implementation meeting to discuss how they could combine these new course recommendations with the ones that the University was already working on merging and developing.

In that meeting, faculty developed the curriculum sequence, and happily agreed to introduce and teach “Nursing Care of the Aging Population” that semester (semester I ; 2019/2020) as a junior level class. Faculty identified students who could take this course since the implementation was important to pick up at that time.

An additional Spreading Change activity involved purchasing and shipping copies of textbooks. Due to funding limitations, strategic decisions identified the most relevant or broad-based books.

Finally, we will continue to monitor and evaluate the Spread of Change of the curricular implementation. One challenge following curriculum dissemination was how to confirm that the new curricula were being implemented with fidelity. Direct observation by LBNM and/or US academic institution faculty would be ideal, and could be incorporated into the accreditation process, but logistically this would be challenging. Travel throughout Liberia to each training institution is not feasible, and timing the observations to match the accreditation process would not allow for timely fidelity checks or ongoing quality assurance, which would be essential to addressing implementation challenges as they occurred.

We therefore elected to review curriculum implementation at each training institution by using course materials as fidelity checks. This allows us to monitor implementation at the classroom level without requiring direct observation or relying only on the self-report of faculty members. We are reviewing course materials at two time points for each class. Prior to the start of the semester, LBNM requests each faculty member to submit their syllabus or course outline. This document is compared to a checklist of topics taken directly from the curriculum. Should discrepancies be identified, they can be addressed immediately and potentially corrected during the semester. At the conclusion of the semester, LBNM requests each faculty member to submit blank copies of all quizzes, exams, and paper and presentation assignments. The contents of these materials, which represent the full range of student assessments in didactic classes, will be compared to the same list of topics taken directly from the curriculum, and this comparison will allow for validation that each topic is adequately covered during the semester. Discrepancies can be addressed and monitored in future semesters. To facilitate this process, we have provided LBNM with a detailed monitoring tool that includes the checklist of topics covered in each course. Each course has a separate tool to allow for easy record keeping and tracking.

This process of reviewing the implementation of the curriculum at the classroom level, which has begun with the October 2020 semester, has several advantages. First, we will be able to determine which faculty and institutions are administering the new curricula with fidelity, and target resources only where they are needed. Second, this methodology provides a framework for LBNM to institutionalize this type of monitoring as part of their overall program oversight responsibilities. Third, this methodology allows us to provide objective feedback to faculty and deans in near real-time, improving the quality assurance process at each institution. Finally, we will be able to assess data from this monitoring at the institution, course, or curriculum level as needed, using databases we will establish for this purpose.

## Lessons Learned/Conclusions

Developing trust and understanding within a large multilateral partnership is critical to the success of the identified, prioritized work. Remote communication is critical, however expectations of reliable access were quickly “right adjusted” with open communication channels.

Because of trusted relationships, when difficult questions needed to be asked, direct and transparent responses were forthcoming. This process eliminated much of the guess work as to how and why actions and processes were accomplished. Our Liberian colleagues are very open with their opinions, which helped us in understanding their culture and developing approaches that worked well with them.

Understanding the infrastructure of Liberia was paramount when considering the work of a national nursing and midwifery curricular revision. Visits by the US academic institutional team members to Liberia, often beyond the capitol city, brought a deeper level of understanding of the strengths and barriers to providing quality nursing and midwifery education in Liberia.

## Next Steps

The LBNM has proven itself a critical RRHS partner that is committed to strengthening the nursing and midwifery health workforce in Liberia. To this end, and to support the continued advancement of nursing and midwifery in Liberia, we are proposing a new investment that will bring Liberia to the forefront of nursing and midwifery in Africa. The US academic institutional partner has already been working with the LBNM to update the national examination licensing questions to reflect new content included in the updated curricula. Through a new tri-partite partnership between the US academic institutional partner, the LBNM, and the Ghanaian Nursing and Midwifery Council (GNMC), we seek to pilot an initiative that will bring the Liberian nursing and midwifery licensing exam to an online electronic platform.

This exciting initiative would significantly advance the efficiency and efficacy of nursing and midwifery licensing in Liberia, and would be the second online licensing examination of its kind in West Africa. Through the West African regional affiliation of nursing and midwifery regulatory boards, and the West African College of Nursing, the LBNM has established a strong relationship with the Ghanaian Council of Nurses & Midwives. In September 2018, Ghana became the first country in West Africa, and the third in Africa, to launch its licensing examination for nurses and midwives online. As a means to streamline and strengthen its operations, the initial pilot was conducted with 375 candidates.

The online process has been touted by all stakeholders, from users to proctors, regulatory board members and the Ministry of Health, as a very positive experience. As evidenced in Ghana, implementation of the examination online has led to significant cost reductions in supplies, printing, and personnel effort, not to mention efficiencies and increased capacity in Information Communication and Technology (ICT) among the country’s nurses and midwives. We look forward to moving forward with the proposed pilot initiative in Liberia.
